# A new *Eimeria* coccidian species (Apicomplexa: Eimeriidae) from Père David’s deer (*Elaphurus davidianus* Milne-Edwards, 1866) in Dafeng Milu National Nature Reserve in Jiangsu Province, eastern China

**DOI:** 10.1186/s12917-022-03308-2

**Published:** 2022-06-03

**Authors:** Weimin Cai, Zeyang Suding, Lele Wang, Zhaofeng Hou, Dandan Liu, Siyang Huang, Jinjun Xu, Jianping Tao

**Affiliations:** 1 College of Veterinary Medicine, Yangzhou University, 12 East Wenhui Road, Yangzhou, Jiangsu 225009 People’s Republic of China; 2 Jiangsu Co-innovation Center for Prevention and Control of Important Animal Infectious Diseases and Zoonoses, Yangzhou University, Yangzhou, 225009 China; 3 Jiangsu Key Laboratory of Zoonosis, Yangzhou University, Yangzhou, 225009 China

**Keywords:** Père David’s deer, Morphology, *Eimeria davidianusi*, Phylogenetics

## Abstract

**Background:**

*Eimeria* coccidiosis is a significant intestinal parasitic disease, which can lead to weight loss, disease and even death of many animals*.* At present, there is no information about the prevalence of *Eimeria* among the world’s endangered species of Père David’s deer (*Elaphurus davidianus*). Therefore, the purpose of this study is to identify an unknown *Eimeria* genus in the Père David’s deer in Dafeng Milu National Nature Reserve, China.

**Results:**

A new *Eimeria* species is described from Père David’s deer. Sporulated oocysts (*n* = 54) are pyriform, with a rough, yellowish brown, 2-layered oocyst wall (2.5 μm thick). A numerous small granules are dispersed randomly on the wall. Oocysts measured 41.2 (39.2–42.8) μm × 29.5 (27.9–30.5) μm, oocyst length/width (L/W) ratio, 1.4. Oocyst residuum, a polar granule and a polar cap are absent. The micropyle (3.5 μm wide) is present. Sporocysts are spindle shaped, 18.2 (16.5–20.0) μm × 10.5 (9.8–11.9) μm, sporocyst L/W ratio, 1.7 (1.5–1.9). A thin convex Stieda body is present and the sporocyst residuum is composed of numerous small granules less than 2.0 μm in diameter dispersed randomly. Each sporocyst contained 2 comma-shaped sporozoites in head-to-tail arrangement. A nucleus is located immediately anterior to the posterior, strong refractive and subspherical refractile body (~ 8 μm). Molecular analysis was conducted at the 18S, ITS-1 and COI loci.

**Conclusion:**

Based on the morphological and molecular data, this isolate is a new species of coccidian parasite, which is named *Eimeria davidianusi* after its host, the Père David’s deer (*Elaphurus davidianus*).

**Supplementary Information:**

The online version contains supplementary material available at 10.1186/s12917-022-03308-2.

## Introduction

**Fig. 1:**
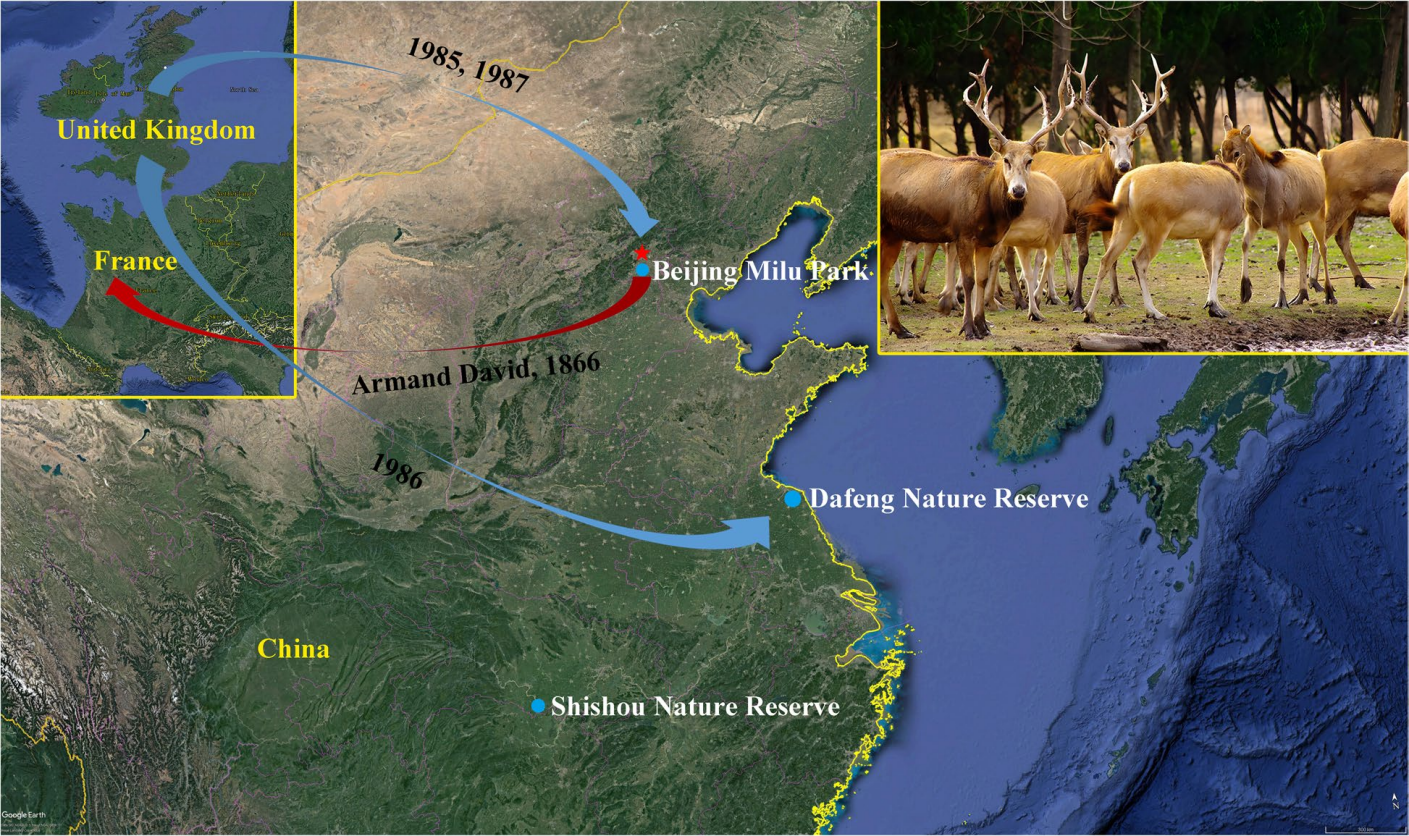
Maple of the decline and rejuvenation of Père David’s deer. The red arrow indicates Père David’s deer were shipped out by Armand David in 1866. The blue arrow indicates Père David’s deer were shipped back in 1985 and 1986

Milu (*Elaphurus davidianus*), also known as Père David’s deer, is a species endemic to China, where it is named “four unlike” because it has a head like a horse, a horn like a deer, a hoof like an ox, and a tail like a donkey [1]. Père David’s deer, originated from the early Pleistocene about 2 million years ago according to the fossils of antlers and bones excavated now [2, 3], are endangered animals in the world [4], and belongs to the genus *Elaphurus* in the family Cervidae [3]. Judging from the historical records of human beings, the Père David’s deer was first seen in the relevant chapters of Mencius of the Zhou Dynasty [5]. Ever since Armand David, a French missionary, first transported Père David’s deer to Europe in 1886, until the early twentieth century, the Père David’s deer population was declared extinct in China [6]. The first reintroduction of 38 Père David’s deer into China have consisted of 2 donations from the Woburn Abbey herd of England. A herd of 20 (5 males, 15 females) in 1985 followed by a herd of 18 female deer in 1987, and both of these herds went to the Beijing Milu Park [3]. In 1986, an additional 39 deer, chosen from 5 zoological gardens in the UK, were given to the Dafeng Milu National Nature Reserve [7], near the Yellow Sea (Fig.1). Today, more than 8000 Père David’s deer are raised in parts of China [8], especially on our nature reserves. According to the report, bacteria [2, 9–11], viruses [12, 13] and parasites such as helminth [14, 15] and protozoa [16–20] are a potential threat to the survival of the Père David’s deer. In order to better protect Père David’s deer, we collected some feces of Père David’s deer for examination, and found an *Eimeria* that had never been described.

More than 1800 species of *Eimeria* have been identified all over the world [21] since Leeuwenhoek found *Eimeria stiedai* in rabbit bile in 1674. Although some studies have been carried out on deer coccidia [22–28], there are few reports on Père David’s deer coccidia so far. At the same time, due to the incomplete description and lack of measurement of many *Eimeria* spp. in the deer family in the past, it is difficult to verify the existing species. As a result of these difficulties, molecular tools [29–34] are essential to accurately delimit species and infer phylogenetic relationships among *Eimeria* species. In the present study, we aimed to: morphologically describe and genetically characterize a novel observed species of *Eimeria* as *Eimeria davidianusi* n. sp. isolate in Père David’s deer (*Elaphurus davidianus*).

## Results

### Description

**Fig. 2:**
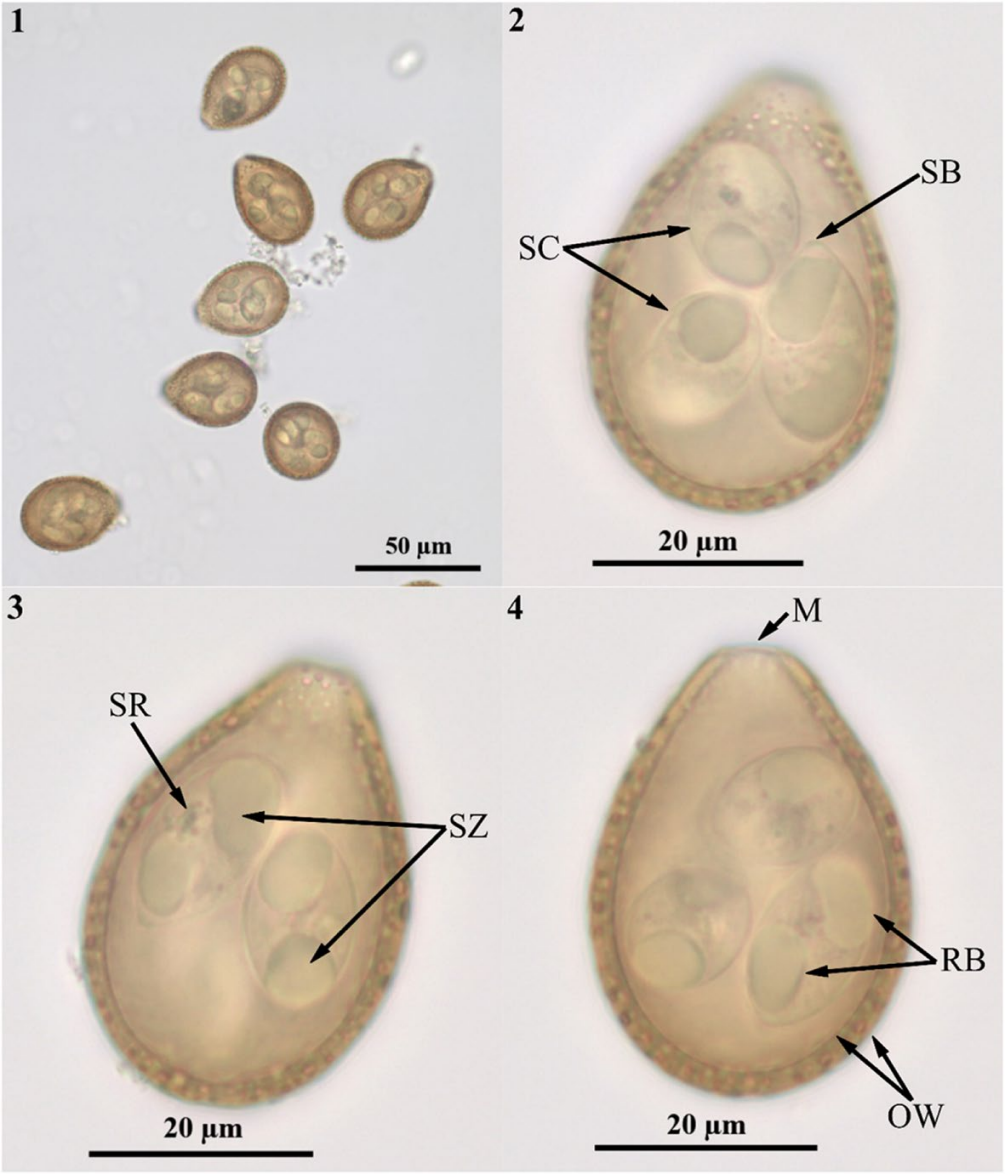
Photomicrographs of the *E. davidianusi* isolate oocysts. 1, 2 and 3 are the visual field of the *E. davidianusi* isolate oocysts under different magnification lenses, respectively. 4, 5 and 6 are the same oocyst’s field of vision under a 100 oil immersion objective. (1 = 10 × objective; 2 = 40 × objective; 3 = 100 × objective; SC = sporocyst; SB=Stieda body; SR = sporocyst residuum; SZ = sporozoties; M = micropyle; RB = refractile body; OW = oocyst wall)

**Fig. 3:**
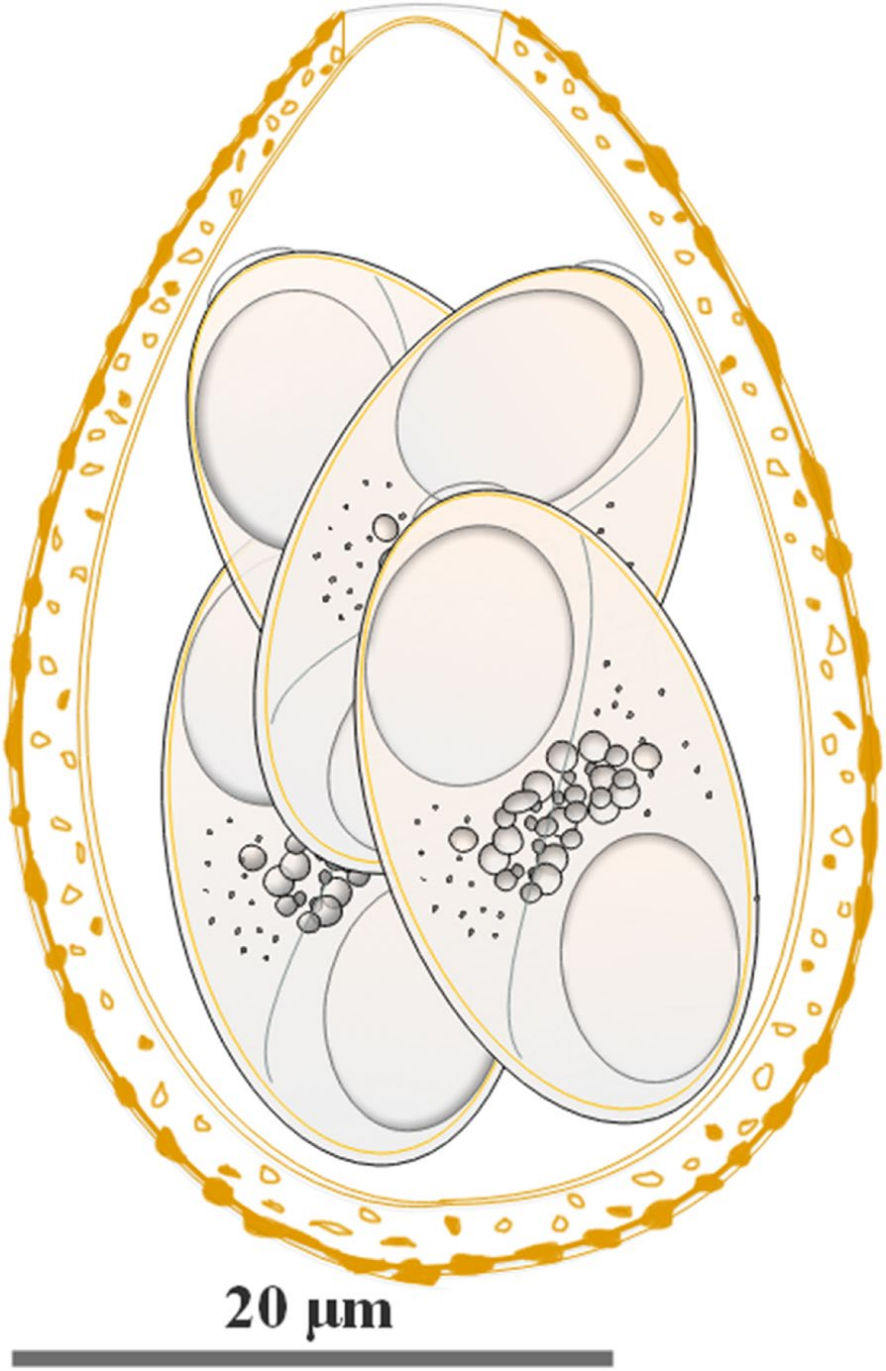
Composite line drawing of the *E. davidianusi* sporulated oocyst. Scale bar = 20 μm

SO (*n* = 54) are pyriform, with a yellowish brown, rough and projecting punctate, 2-layered oocyst wall (2.5 thick). Oocysts measured 41.2 (39.2–42.8) × 29.5 (26.5–30.6), oocyst length/width (L/W) ratio, 1.4. OR, PG and PC are absent. The M (3.5 wide) is present. SC are spindle shaped, 18.2 (16.5–20.0) × 10.5 (9.8–11.9), SC L/W ratio, 1.7 (1.5–1.9). A tiny flattened SB is present, SSB is absent and the SR is composed of numerous small granules less than 2.0 in diameter dispersed centrally (Table 1). Each SC contained 2 comma-shaped SZ in head-to-tail arrangement (Fig. 2). One end of the SZ has a large spheroidal posterior refractile body and a nucleus that does not appear to be very clear, which is located in the center of the SZ. A more concise picture of the oocyst pattern is shown in Fig. 3.

**Table 1:** Comparative morphology of *E. davidianusi* from Père David’s deer with other *Eimeria* species recorded from ruminants and the pig

Species	Hosts	Oocyst							Sporocysts					References
		Shape	Size (μm)	Shape Index	Wall (thick/μm)	Micropyle (wide/μm)	Polar Granule	Oocyst Residuum	Shape	Size (μm)	Shape Index	Stieda Body	Sporocyst Residuum	
*E. bukidnonensis*	Cattle	Pyriform	47.4 × 33 (43–51 × 30–35)	1.44	2-layered (3.5)	Present	Absent	Absent	Elongate	19.6 × 9.8 (18–21 × 9–11)	2.00	Present	Absent	Courtney et al., 1976 [35]
*E. wyomingensis*	Cattle	Pyriform	39.9 × 28.3 (36–44 × 26–30)	1.41	2-layered (2.5)	Present	Absent	Absent	Elongate	18.7 × 8.6 (17–20 × 8–10)	2.17	Present	Absent	Courtney et al., 1976 [35]
*E. alabamensis*	Cattle	Pyriform	19.5 × 14.3 (16–25× 12–17)	1.36	1-layered (0.5)	Absent	N/A	Absent	Elongate	8.2 × 3.8	2.16	Tiny	Tiny	Christensen, 1941 [36]
*E. subspherica*	Cattle	Subspherical	11.5 × 10.6 (9–13 × 8–12)	1.08	N/A	Absent	N/A	Absent	Elongate	8.1 × 3.6	2.25	Tiny	Absent	Christensen, 1941 [36]
*E. macusanieniensis*	Alpaca	Ovoid to pyriform	93.6 × 67.4 (81–107 × 61–80)	1.39	3-layered (8–12)	Prominent (9–14)	Absent	Absent	Elongate to ovoid	36.3 × 18.3 (33–40 × 16–20)	1.98	Faintly perceptible	Faintly perceptible	Guerrero et al., 1971 [37]
*E. intricata*	Sheep	Ellipsoidal to ovoid	50 × 38 (39–59 × 27–47)	1.32	2-layered (2.4–3.8)	Present	Present	Absent	Elongate to ovoid	17–22 × 9–14	N/A	Absent	Present	Hao, 2017 [38]
*E. mayeri*	Reindeer	Spheroidal to ellipsoidal	17.2 × 14.1 (9–21 × 9–16)	1.22	2-layered (1)	Present (<1)	Absent	Absent	Ovoid	9.3 × 5.1 (8–11 × 4–6)	1.82	Nipple-like	Inconspicuous	Gudmundsdottir and Skirnisson, 2005 [39]
*E. rangiferis*	Reindeer	Ovoid	34.9 × 27.6 (31–38 × 25–30)	1.26	2-layered (1.8–2.0)	Present (4–6)	Absent	Absent	Spindle shaped	18.6 × 9.2 (17–20 × 8.0–10)	2.02	Nipple-like	Present	Gudmundsdottir and Skirnisson, 2005 [39]
*E. hreindyria*	Reindeer	Ellipsoidal	30.0 × 21.1 (24–35 × 18–23)	1.42	2-layered (0.8–1.2)	Absent	Present	Absent	Spindle shaped	15.3 × 6.5 (13–18 × 6–8)	2.35	Present	Present	Gudmundsdottir and Skirnisson, 2006 [40]
*E. alces*	Elk	Ovoid	38.0 × 26.1 (33–43 × 24–29)	1.46	2-layered (2.2)	Distinct (~7)	Present	Absent	Ovoid to elongate	18.6 × 8.9 (17.0–21 × 8–10)	2.09	Present	Absent	Pyziel and Demiaszkiewicz, 2013 [24]
*E. cervi*	Sika deer	Pyriform	35.2 × 25.1 (26–39 × 20–29)	1.40	2-layered (0.6–1.0)	Prominent (4.2)	Absent	Absent	Long oval	18.2 × 9.7	N/A	Present	Present	Lu, 1995 [41]
*E. robusta*	Sika deer	Ovoid	35.3 × 26.1 (27–41 × 21–30)	1.35	2-layered (0.6–1.1)	Prominent (5.8)	Absent	Absent	Long oval	18.2 × 9.6	N/A	Present	Present	Lu, 1995 [41]
*E. sordida*	Sika deer	Ellipsoidal to ovoid	34.3 × 25.6 (30–38 × 21–28)	1.34	2-layered (0.4–1.1)	Prominent (5.8)	Absent	Absent	Long oval	20.1 × 8.9	N/A	Present	Present	Lu, 1995 [41]
*E. austriaca*	Sika deer	Ellipsoidal to ovoid	23.3 × 20.0 (19–26 × 15–23)	1.16	layered (0.4–1.1)	Absent	Absent	Absent	Long oval	10.7 × 6.4	N/A	Absent	Absent	Lu, 1995 [41]
*E.* sp-1	Sika deer	Subspherical	28.0 × 26.3 (24–31 × 21–30)	1.07	2-layered (0.3–1.1)	Absent	Absent/only one	Absent	ovoid	15.0 × 8.6	N/A	Present	Present	Lu, 1995 [41]
*E.* sp-2	Sika deer	Ellipsoidal	33.4 × 25.7 (27–38 × 20–28)	1.30	2-layered (0.5–1.0)	Absent	Absent	Absent	Long oval	19.1 × 10.0	N/A	Absent	Present	Lu, 1995 [41]
*E. scabra*	Pig	Ovoid to subspherical	25–45 × 18–28	N/A	2-layered (1.5–3)	Present	Present	Absent	Ovoid	14–18 × 7–9	N/A	Prominent	Present	Levine, 1985 [42]
*E. davidianusi*	Père David's deer	Pyriform	41.2×29.5 (39-43×26-31)	1.40	2-layered (1.5–2.9)	Prominent (2.8–4.0)	Absent	Absent	Spindle shaped	18.2×10.5 (16-20×10-12)	1.73	Present	Present	Present study

*Host*: Père David’s deer (*Elaphurus davidianus*, Milne-Edwards, 1866).

*Locality*: Dafeng Milu National Nature Reserve (32°56′- 33°36′ N ~ 120°42′- 120°51′ E), eastern China.

*Prevalence*: 1 / 1(100%).

*Other hosts*: Unknown.

*Prepatent period*: Unknown.

*Patent period*: Unknown.

*Site of infection*: Unknown.

*Sporulation time*: 120–144 h.

Material deposited: the 18S, ITS-1 and COI sequences were submitted to GenBank, and the accession number were MT822711, MT822712, and MT822713.

Etymology: This species is named *Eimeria davidianusi* after its host.

### Phylogenetic analysis of *E*. *davidianusi* at the 18S locus

**Fig. 4:**
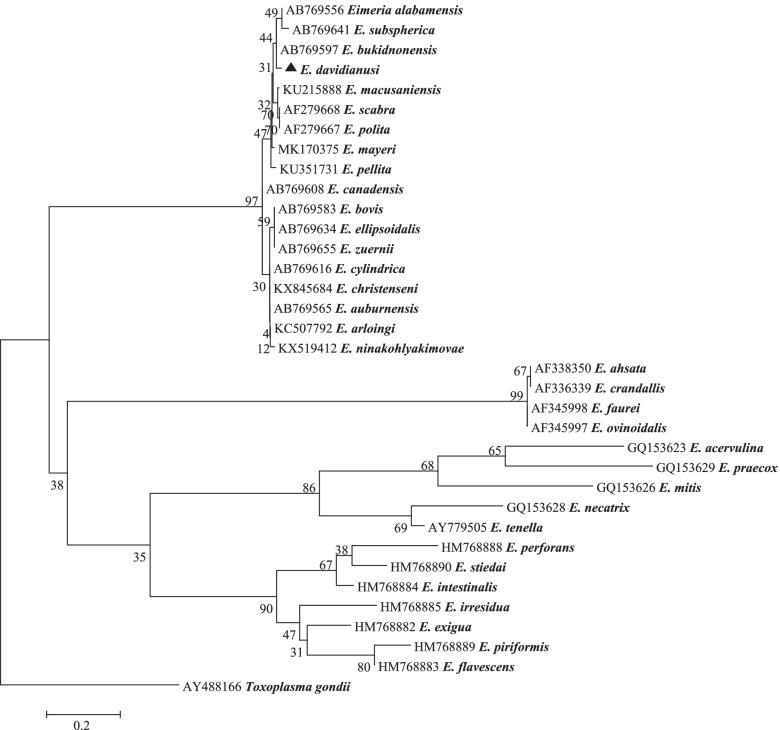
Evolutionary relationships of *E. davidianusi* inferred by distance analysis of 18S rRNA sequences (1380 bp). Percentage support from 1000 pseudoreplicates from Neighbor-joining (NJ) analysis is indicated at the left of the supported node

At the 18S rRNA locus, a 1380 bp PCR product of *E. davidianusi* isolate was successfully amplified and sequenced. Phylogenetic analysis of *E. davidianusi* isolate at this locus using Distance, ML and NJ analyses produced similar results (Fig. 3). There are no 18S rRNA sequences from *Eimeria* derived from Père David’s deer available in GenBank, therefore phylogenetic analysis could only be conducted using available *Eimeria* 18S rRNA sequences. *Eimeria davidianusi* grouped in a separate clade. It shared 97.76 and 97.69% genetic similarity with *E. alabamensis* (Christensen, 1941) (AB769556) and *E. bukidnonensis* (Tubangui, 1931) (AB769597) from cattle in Japan (Fig. 3). It exhibited 97.47% genetic similarity to *E. faurei* (Moussu and Marotel, 1901) (AF345998), which was identified from a sheep from Turkey. *Toxoplasma gondii* (Lave ran, 1900) (AY488166) was used as an outgroup (Fig. 4).

### Phylogenetic analysis of *E*. *davidianusi* at the ITS-1 locus

**Fig. 5:**
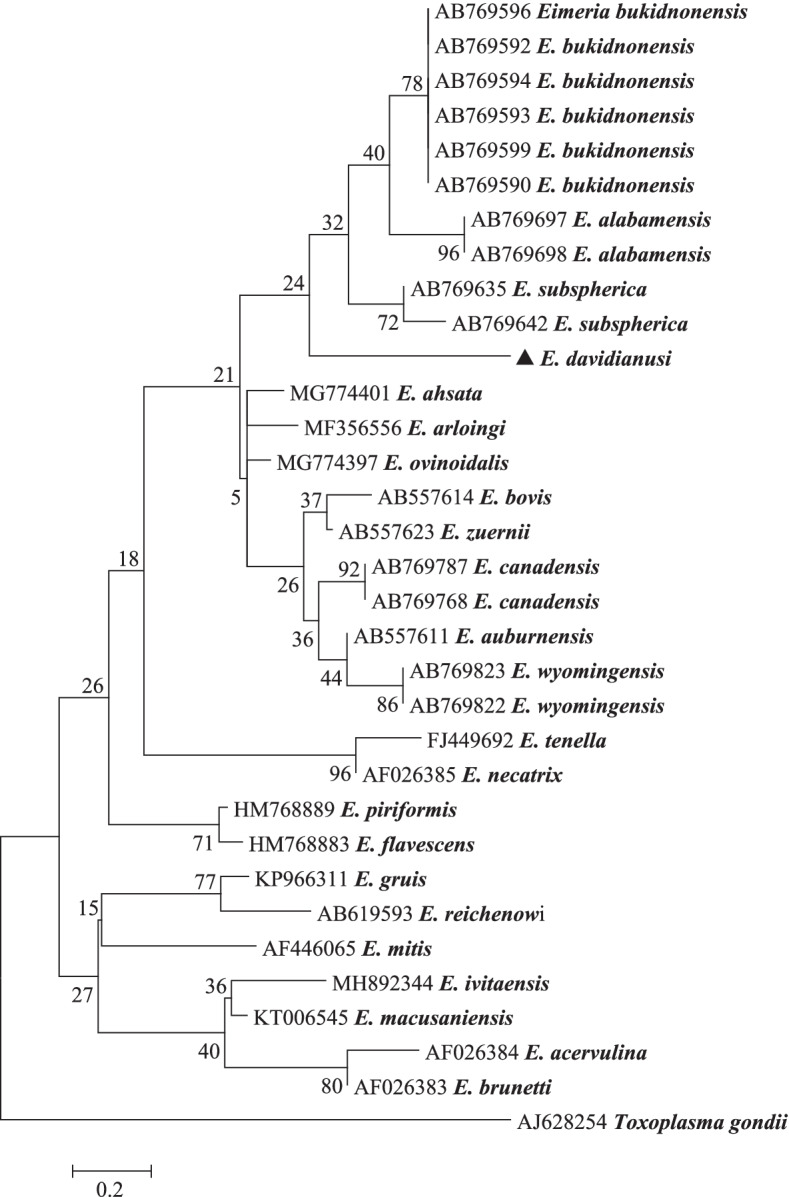
Evolutionary relationships of *E. davidianusi* inferred by distance analysis of ITS-1 sequences (328 bp). Percentage support from 1000 pseudoreplicates from Neighbor-joining (NJ) analysis is indicated at the left of the supported node

A 328 bp sequence of ITS-1 from *E. davidianusi* was used for phylogenetic analysis. There are no ITS-1 sequences from *Eimeria* derived from Père David’s deer available in GenBank, therefore phylogenetic analysis could only be conducted using available *Eimeria* ITS-1 rRNA sequences. *Toxoplasma gondii* (AJ628254) was used as an outgroup. Phylogenetic analysis grouped the *E. davidianusi* isolate in a separate clade and shared 97.50 and 96.38% genetic similarity with *E. bukidnonensis* (AB769599) and *E. subspherica* (Christensen, 1941) (AB769642) from cattle in Japan (Fig. 5).

### Phylogenetic analysis of *E*. *davidianusi* at the COI locus

**Fig. 6:**
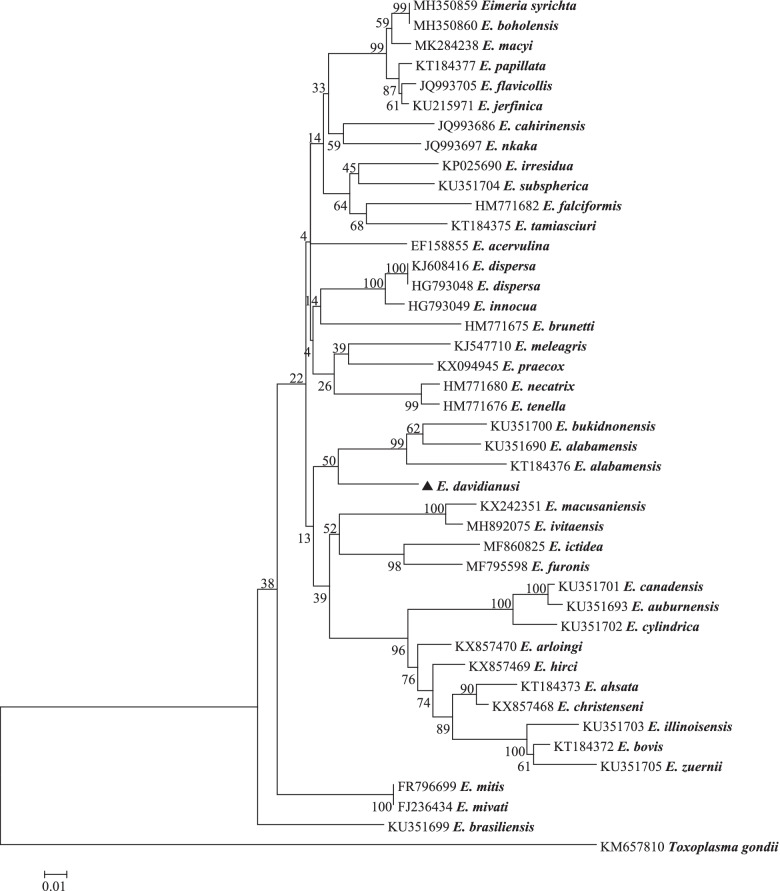
Evolutionary relationships of *E. davidianusi* inferred by distance analysis of COI sequences (786 bp). Percentage support from 1000 pseudoreplicates from Neighbor-joining (NJ) analysis is indicated at the left of the supported node

Phylogenetic analysis of the 786 bp COI sequence placed *E. davidianusi* in a clade with *E. bukidnonensis* (KU351700) and *E. alabamensis* (KU351690, KT184376) (50.0% similarity). There are no COI sequences from *Eimeria* derived from Père David’s deer available in GenBank, therefore phylogenetic analysis could only be conducted using available *Eimeria* COI sequences. It exhibited 90.29% genetic similarity to *E. bukidnonensis* (KU351700), which was identified from cattle from Turkey. *Toxoplasma gondii* (KM657810) was used as an outgroup (Fig. 6).

## Discussion

Traditionally, identification of *Eimeria* species has been based largely on sporulated oocyst morphology and some biological characteristics such as pathological changes, incubation period, sporulation time [22–25]. In practice, a small portion of oocysts may differ from the expected typical morphology, and all these biological characteristics can present a variable level of overlap, making it difficult in some cases to identify *Eimeria* species accurately. In view of the limitations of microscopic identification, molecular techniques have been developed as a method of detection and specific identification of species of the genus *Eimeria* [43–47]. For this reason, we used three genes (18S rRNA, ITS-1 and COI) as molecular markers to ensure the accuracy of identification and phylogeny of the new *Eimeria* species in this paper.

The family Cervidae within the Artiodactyla includes 19 genera with 51 species of deer (http://www.departments.bucknell.edu/biology/resources/msw3/browse.asp?s=y&id=14200205). Based on the mitochondrial and nuclear phylogenies of Cervidae, *Elaphurus* is most closely related to the genus *Cervus* [48]. Currently, 37 *Eimeria* species have been named from the family Cervidae, of which 11 species, including *E. elaphi* (Jansen and van Haaften, 1966), *E. austriaca* (Supperer and Kutzer, 1961), *E. wapiti* (Honess, 1955), *E. zuernii* (Rivolta, 1878), *E. sordida* (Supperer and Kutzer, 1961), *E. cervi* (Galli-Valerio, 1927), *E. robusta* (Supperer and Kutzer, 1961), *E. asymmetrica* (Supperer and Kutzer, 1961), *E. gallivalerioi* (Rastegaieff, 1930), *E. hegneri* (Rastegaieff, 1930) and *E. schoenbuchi* (Boch, 1963) were described from genus *Cervus* (https://www.k-state.edu/parasitology/worldcoccidia/CERVIDAE). In China, 6 *Eimeria* species, including *E. austriaca*, *E. cervi*, *E. robusta*, *E. sordida* and two indeterminate species had been reported from sika deer [41], but their morphology is different from this new species (Table 1). The new species, *Eimeria davidianusi*, represents the first coccidian species described from the Père David’s deer.

The oocyst morphology of *E. davidianusi* is very similar to that of *E. bukidnonensis*, a species of bovine coccidia. The oocysts of *E. bukidnonensis* were pyriform and measured 47.4 (43–51) × 33.0 (30–35); sporocysts were 19.6 (18–21) × 9.8 (9–11). M is present (about 3–5 in diameter) and PG, OR, and SR are absent. Oocyst wall of 2 layers, 3.5 thick, and dark brown [35]. In phylogenetic relationships of two species, the pairwise genetic distance of *E. davidianusi* and *E. bukidnonensis* at the 18S rRNA, ITS-1 and COI locus is 97.69, 97.50 and 90.29%, respectively, and phylogenetic analysis revealed that *E. davidianusi* was closely to *E. bukidnonensis*. Therefore, we speculated that *E. davidianusi* and *E. bukidnonensis* may evolve from a common ancestor that parasitized some ancient ancestor of deer and cattle, thus co-speciating with their respective hosts, while still maintaining plesiomorphic features. Surprisingly, the existing research results suggest that Père David’s deer and cattle may have a common ancestor, such as ancient deer or ancient cattle [49].

Similar to oocysts of *E. davidianusi*, *E. wyomingensis* (Huizinga and Winger, 1942) from cattle, *E. intricata* (Spiegl, 1925) from sheep, *E. macusaniensis* (Hernandez, Bazalar and Alva, 1971) from alpacas and *E. scabra* (Henry, 1931) from pigs have a rough wall and a micropyle (Table 1), but these species differ greatly from *E. davidianusi* in molecular characteristics. On the contrary, *E. alabamensis* from cattle and *E. subspherica* from cattle are similar to *E. davidianusi* in molecular characteristics but far apart in morphology (Table 1). These results are consistent with Ogedengbe’s findings [50]. Owing to no sequences of 18S, ITS-1 and COI of *Eimeria* from the family Cervidae in GenBank, the phylogenetic relationships between Cervidae coccidia and this new species couldn’t be analysed.

## Conclusion

In summary, this is the first report of the morphological and molecular characterization of an *Eimeria* sp. in Père David’s deer worldwide. A new *Eimeria* coccidian species (Apicomplexa: Eimeriidae) from Père David’s deer in Dafeng National Nature Reserve in eastern China has been identified which is named *E. davidianusi*.

## Materials and methods

### Sample collection

In May 2018, a juvenile Père David’s deer occurred diarrhea in Dafeng Milu National Nature Reserve, Jiangsu Province. We collected fecal samples and stored them in an insulated field box until processed, which was no later than 5 hours after collection. Shortly after returning to the laboratory and microscopy revealed unsporulated coccidian oocysts.

Fecal flotation was conducted using a saturated sodium chloride and 50% sucrose (w/v) solution [51]. A portion of feces was placed in 2.5% (w/v) potassium dichromate solution (K_2_Cr_2_O_7_) [52], mixed well and poured into Erlenmeyer flasks (250 mL) to a depth of less than 1 cm and kept at room temperature in the dark to facilitate sporulation.

### Morphological analysis

Fifty-four sporulated *Eimeria* spp. oocysts of a consistent, novel morphology (obtained from a Père David’s deer) were observed using a Carl Zeiss AxioCam ICc 5 (Jena, Germany) digital microimaging camera and photographed with a 100× oil immersion objective. It was observed that there was only one species of coccidial oocysts in the fecal samples. Images were analyzed using ZEN 2012 (blue edition) software, to obtain measurements of oocyst length and width, oocyst wall thickness and sporocyst length and width. Due to the compacted nature of this species of *Eimeria*, measurements were only taken from one sporocyst per oocyst and the sporocyst was subjectively identified as being positioned laterally. Where no sporocysts could be manipulated into lateral position within the oocyst, sporocyst length measurement was not taken. The oocysts were compared with some published coccidia of Artiodactyla to observe the morphological similarities and differences [24, 35, 37–42].

### DNA isolation

Six hundred oocysts were ground in liquid nitrogen for five times. Grinded oocyst fragments were transferred to a 1.5 mL centrifugal tube and the remaining operations were performed according to MiniBEST Universal Genomic DNA Extraction Kit Ver.5.0 (TaKaRa Biomed, Beijing, China) instructions for DNA extraction (https://www.takarabiomed.com.cn/ProductShow.html).

### PCR amplification

A standard PCR with the primers E18SF and E18SR (Table 2) [47] was used for amplification of the 18S ribosomal RNA (abbreviated 18S rRNA) gene. The expected PCR product was ~ 1500 bp. The PCR reaction (50 μL) were performed in 1 μL (10–20 ng) of genomic DNA, 10 pM of each primer and 2.5 U Premix Taq polymerase (TaKaRa, Tokyo, Japan) in a thermocycler (Bio-Rad, CA, USA) under the following conditions: 94 °C for 4 min (initial denaturation), followed by 30 cycles of 94 °C for 60 s (denaturation), 59 °C for 45 s (annealing), 72 °C for 60 s (extension), and then a final extension of 72 °C for 10 min.

**Table 2:** Sequences of primers

Name of primer	Sequence (5' to 3')
For 18S rRNA	
E18SF	GAAACTGCGAATGGCTCATT
E18SR	CTTGCGCCTACTAGGCATTC
For ITS1	
EIF	AAGTTGCGTAAATAGAGCCC
EIR	CAAGACATCCATTGCTGAAA
For COI	
ECOIF	GTTTGGTTCAGGTGTTGGTTGGAC
ECOIR	ATCCAATAACCGCACCAAGAGATA

The PCR for the first internal transcribed spacer (ITS-1) locus was carried out with the primers EIF and EIR (Table 2) [53]. The expected PCR product was ~ 450 bp. The PCR reaction contained 2.5 U Premix Taq polymerase (TaKaRa, Tokyo, Japan), 10 pM of each primer and 1 μL (10–20 ng) of genomic DNA. The PCR was conducted using the following cycling conditions: 1 cycle of 94 °C for 4 min, followed by 30 cycles of 94 °C for 60 s, 56 °C for 30 s and 72 °C for 60 s and a final extension of 72 °C for 10 min.

A partial cytochrome c oxidase subunit I (COI) gene sequence was amplified using a standard PCR with the following primers ECOIF and ECOIR (Table 2) [54]. The expected PCR product was ~ 900 bp. The PCR reaction (50 μL) were performed in 1 μL (10–20 ng) of genomic DNA, 10 pM of each primer and 2.5 U Premix Taq polymerase (TaKaRa, Tokyo, Japan) in a thermocycler (Bio-Rad, CA, USA) under the following conditions: 94 °C for 5 min (initial denaturation), followed by 30 cycles of 94 °C for 60 s (denaturation), 50 °C for 30 s (annealing), 72 °C for 60 s (extension), and then a final extension of 72 °C for 10 min.

### Sequence analysis

Samples without DNA (no-DNA controls) were included in each amplification run, and in no case were amplicons detected in the no-DNA controls. Each amplicon (10 μL) was examined by agarose (1%) gel electrophoresis, stained with ethidium bromide and photographed using a gel imaging system (Bio-Rad, CA, USA). All PCR products yielded a single band and were purified by MiniBEST DNA Fragment Purification Kit Ver.4.0 (TaKaRa, Tokyo, Japan). Purified PCR products were sent to GenScript (Nanjing, China) for sequencing from both directions by using a primer walking strategy.

The results of the sequencing reactions were analyzed and edited using DNAstar software, compared to existing *Eimeria* sp. 18S, ITS-1 and COI sequences on GenBank using BLAST searches and aligned with reference genotypes from GenBank using Clustal W in MegAlign and MAFFT (https://www.ebi.ac.uk/Tools/msa/mafft/).

### Phylogenetic analysis

Phylogenetic trees were constructed for *Eimeria* sp. at the 18S, ITS-1 and COI loci with additional isolates from GenBank. Parsimony analyses were conducted using MEGA (Molecular Evolutionary Genetics Analysis software, version 5, Arizona State University, Tempe, Arizona, USA). Neighbor-joining (NJ) and maximum likelihood (ML) analyses were conducted using Tamura-Nei based on the most appropriate model selection using ModelTest in MEGA 5 [55]. Bootstrap analyses were conducted using 1000 replicates to assess the reliability of inferred tree topologies.

### Statistical analysis

Measurements of 54 sporulated oocysts were analyzed using Statistical Package for the Social Sciences (SPSS Version 22) and results are presented in micrometres as the mean, with the observed range in parentheses. Since all measurement units in the article are microns, all length units except those in the summary have been omitted in order to follow the standardized format.

### Line drawing

The oocyst line drawing was constructed using the software of Edraw Max (https://www.edrawsoft.cn/).

## Supplementary Information

**Additional file 1.**

## Data Availability

The datasets generated and/or analysed during the current study are available in the GenBank repository (MT822711 to MT822713). All data and additional files are available from the corresponding author on reasonable request.

## Abbreviations

OR: oocyst residuum
PG: polar granule
SSB: sub Stieda body
18S: 18S ribosomal RNA
ITS-1: internal transcribed spacer 1
COI: cytochrome c oxidase subunit I
